# Clinical Notes

**Published:** 1888-11

**Authors:** E. W. Berridge


### CLINICAL NOTES.

(1) *Indium metallicum.*—January 9th, 1888.—A boy, ten years old, had suffered for five or six years with constipation. Some remedies in high potencies had been given him, but with only temporary benefit. He had had no medicine for a very long time when I prescribed *Indium.* Present symptoms : stool about once a week, dark and thick ; sometimes with blood ; anus sore after stool. Has to strain much, seizing his thighs with his hands and straightening himself forcibly ; the effort makes his face red *and the head feels as if it would burst.* Four doses of *Indium metallicum*$^{cm}$ (Fincke) and one of 60m (Fincke) cured him. Each dose was allowed to act till its effect seemed exhausted, the symptoms beginning to return and persisting.

(2) *Hæmatoxylon.*—July 9th, 1887.—Miss E., aged forty-five.

Has nearly lost voice from getting overheated. It began with hot, sore patch in larynx yesterday afternoon. This morning, on waking, could scarcely breathe. Ever since she can remember has been subject to throat attacks from the least damp, and the slightest cold always attacks this part. These attacks last for a week, badly, and continue less severely for two or three weeks more, her voice not returning fully till the end of this time. Present symptoms: soreness of throat on swallowing saliva. *Feeling of a bar across centre of chest* about level of clavicles; the bar feels heavy and hot and is very burning on waking in morning; it feels like a solid, square bar with sharp edges. She always has this "bar" symptom in these attacks and later there is a feeling of fluttering of a feather there, causing constant, irritating cough, which does not relieve it; but this symptom has not yet had time to come on. She has to fetch the breath by an exertion over the "bar." Both her parents died of phthisis; her two sisters are very rheumatic.

*Hœmatoxylon*$^{200}$, a dose at once, and every three hours till better.

July 14th.—Reports very much better. After the fourth dose the "bar" feeling was less; it felt less heavy and with less sharp an edge, and the smarting and soreness were more diffused over chest. Her voice has now returned. On the night after I prescribed for her the upper part of throat felt inflamed and looked inflamed and glazy. The "bar" feeling had quite gone by evening of July 10th; at the same time her cough became looser and the nose began to run, which it very seldom does in these attacks. The feather feeling hardly came on at all. She says the medicine has cut the attack short.

August 2d.—Reports that she soon recovered. She has had one other cold, but it quickly ceased without medicine.

February 29th, 1888.—Has not had a bad cold since, till now, when there was excessive irritation in upper throat, worse evening and night; feeling of great weakness in throat; hoarseness, and a threatening of the "bar" feeling last night.

I gave one dose of *Hœmatoxylon*$^{5m}$ (F. C.), prepared by my friend, Dr. Tyrrell, of Toronto, and it soon cured.

(3) *Cajeput.*—May 23d, 1887.—Miss B., aged twenty-one. For a week or more, tongue has felt swollen, *seeming to fill the whole of mouth*, and making her lisp. Having no higher potency, and as Dr. Skinner had refused to send me any more of his potencies, and there was no time to write to Dr. Tyrrell for one, I gave her a dose of *Cajeput*$^{30}$, telling her to take a daily dose till better. She continued the medicine too long and experienced the following proving:

May 24th.—On waking this morning, tongue was very dry and felt shrunk to rather less than its natural size—a contracted, dry feeling; when it became moist it gradually assumed its former condition. (This is new.)

June 10th.—She took a daily dose till May 30th. Every morning, on waking, she had the same symptoms as on May 24th, till she stopped taking it. Yesterday the tongue, which had got quite well, began to feel too large again. After taking the medicine for three days, felt low-spirited, thoroughly miserable, also irritable, but this now less.

I gave her one dose of *Cajeput*$^{200}$. It made her feel more depressed than ever, sensitive if any one spoke sharply to her. Tongue got quite well.

(4) *Carlsbad.*—June 16th, 1885.—General H., aged sixty-two, had been under my treatment for two months for sciatica with great benefit. To-day he complained of cramp last night in left ankle; formerly has had it in both; it comes on about five or six A. M., wakening him; it extends down to instep and up to a little above ankle. As he had had no medicine for a month, and, in addition to this new symptom, the old hip pain had begun to return, I concluded the first medicine (*Sepia*) had finished its work, and I now gave him *Carlsbad* (*Sprudel*) 2m (F. C.), a dose in water twice daily for fourteen days.

July 18th reports that he soon improved in every way, and that for ten days has been quite free from the cramp. *No medicine.*

July 29th.—He reported that he felt paralyzed in loins for several hours after rising from bed, better as the day advanced.

This symptom he never had before, and it very much resembles the provings of *Carlsbad:* a very hot hip-bath would partially remove it. No return of the cramp. Mineral springs are exceedingly powerful remedies, and need a thorough proving. In 1885 a lady patient complained of cramps in the fourth and fifth toes of right foot, and told me that while drinking the waters at Kissengen they were much worse than they had ever been.

(5) *Onosmodium Virginianum.*—In Bœricke & Tafel's *Bulletin* for November, 1885, is published a corrected and enlarged version of Dr. Green's provings, with comments by Dr. S. A. Jones.

On February 26th, 1886, a man, aged thirty-eight, consulted me about sexual weakness, arising from bad habits in youth. I had previously removed his headaches after nocturnal emissions with *Conium* $^{cm}$ (F. C.). He has taken no medicine for seven months. He now consulted me again and I gave him *Onosmodium Virg.* twice daily, at first in the ninth centesimal potency (having no higher), and afterward, as soon as I could obtain it, Dr. Swan's DMM potency. It did him much good; the nocturnal emissions, which occurred about five times a month, were reduced to two monthly, and did not exhaust him so much, nor was he so sleepless afterward. There was less escape of semen after sexual excitement. Formerly any sexual excitement would cause a semi-erection and a very slightly painful feeling in penis, which would feel uncomfortably large; this was entirely removed. His condition of impotence was not affected by the remedy as far as I know, for he ceased treatment after six months. Perhaps it was just as well that he should not have been restored to virility, for his father and brother had died in a lunatic asylum and a sister died of phthisis. For such a man to beget children is a crime against humanity.

In another case of sexual weakness, the ninth potency removed a too speedy emission, with deficient erection and diminished pleasure; coldness of glans penis; failure of memory; irritability; unsteadiness of legs while walking.

On February 12th, 1886, I began to prove the medicine on myself, being in good health. I took the ninth potency twice daily. The only symptom was, on the first night I did not sleep well all night; restless, turning from side to side, or moving limbs constantly; slept little, waking often. A little before four A. M., woke on left side with a sort of nightmare, dreaming about fighting with dynamiters; slept a little better afterward, but not well.

This remedy deserves a more extensive proving.

The balance of the evening was taken up with a novel feature in the form of a materia medica quiz, which, from its great success, is likely to prove a prominent feature in future meetings. It was taken part in by all present and generally acknowledged as a very profitable innovation.

Adjourned at a late hour.

WM. JEFFERSON GUERNSEY, *Secretary.*

---

WHAT IS THE REMEDY?—"In response to this inquiry in your last issue, I hasten to reply, Kalmia."—F. POWELL.

